# Longitudinal Effects of Excessive Weight and Obesity on Academic Performance of Primary School Boys in Different Socio-Economic Statuses: The NW-CHILD Study

**DOI:** 10.3390/ijerph18178891

**Published:** 2021-08-24

**Authors:** Dané Coetzee, Wilmarié du Plessis, Deidré van Staden

**Affiliations:** Physical Activity, Sport and Recreation (PhASRec), Focus Area, Human Movement Sciences, Faculty of Health Science, North-West University, Private Bag X6001, Potchefstroom 2520, South Africa; 20376138@nwu.ac.za (W.d.P.); deidrevanstaden@gmail.com (D.v.S.)

**Keywords:** academic performance, boys, obesity, overweight, primary school

## Abstract

Obesity affects millions of children worldwide and can often impact their academic performance. This longitudinal study, conducted over seven years, determines the effects of excessive weight and obesity on the academic performance of primary school boys, taking into account their socio-economic status (SES). The study forms part of a seven-year (2010–2016) longitudinal study, the North-West Child-Health-Integrated-Learning and Development (NW-CHILD) study, which includes a baseline measurement and two follow-up measurements of the 181 participants from varying areas in the North West Province. Two-way frequency tables, repeated measure ANOVA’s and Spearman rank order correlations were used to analyze the data. The Body Mass Index (BMI) of the participants reported an increase from 2010–2016. Nearly all of the school subjects reported small to large correlations between BMI and academic performance (*r* ≥ 0.1 and *r* ≥ 0.3), except for Afrikaans in 2013 (*r* = −0.06). Only two subjects (English and Language as tested with the ANA test) reported medium effects (*r* ≥ 0.3), whereas the other subjects only reported small effects (*r* ≥ 0.1). No statistically significant relationships (*p* ≥ 0.05) were observed between the BMI values and academic subjects, however SES and school subject scores reported several statistically significant relationships, especially regarding Language (English and First Additional Language) and Mathematics (*p* = 0.02). Overweight and obese primary school boys in the North West Province of South Africa reported a higher academic performance in comparison to boys of a normal weight, even when SES was taken into consideration. Further studies are recommended to verify current findings regarding weight, obesity and academic performance.

## 1. Introduction

Forty-two million children (under the age of five years) worldwide are overweight and obese, a global occurrence which affects both children and adults [1,2]. Being overweight or obese usually relates to excessive or abnormal accumulation of fat in the adipose tissue, which may lead to health problems [3]. The prevalence of excessive weight and obesity is a problem both locally (South Africa) and internationally.

Various researchers have confirmed a high prevalence of excessive weight and obesity among boys between the ages of 5 and 18 years in various parts of the world [4,5,6]. These researchers have also reported a prevalence of excessive weight among boys ranging from 11.8%–16.33%, while the prevalence of obesity was slightly lower, ranging from 4.9%–10.69%. Zhang and Wang [7] conducted a trend analysis and reported that the prevalence of excessive weight in boys aged between 7 and 18 years old in Shandong, China, increased from 6.5% in 1995 to 19.1% in 2010 and for obesity from 1.6% to 9.3% in the same period. Several longitudinal studies have reported the increase in the prevalence of excessive weight and obesity over time [8,9]. In this regard, Chen et al. [8] reported that the prevalence of overweight/obese Taiwanese boys increased over the period of six years (from 18.8% at baseline to 35.0%). Furthermore, Cunningham et al.’s [10] longitudinal study in the United States of America (USA) on children aged between 5 and 14 years indicated that the prevalence of excessive weight and obesity (1.9%) was lower among the older boys, while the prevalence of overweight or obese kindergarten boys (13.7%) was much higher. These researchers also found that if the boys were overweight at the age of five years, they are four times more likely to become obese, compared to children of a normal weight. In contrast, another longitudinal study conducted by Ells et al. [9] on English children aged between 4 and 5 and 10 and 11 years old reported that the younger threshold had a lower prevalence of obesity (9.7%) compared to the older (20.4%). With regard to race, weight and obesity, various studies from the USA have reported that African children experience a higher prevalence of excessive weight and obesity (ranging from 11.1% to 27.2%) when compared to Caucasian children (ranging from 5.5% to 17.94%) [11,12,13,14]. Not only is race a contributing factor to the prevalence of excessive weight and obesity, but so is SES. Researchers in the USA and the United Kingdom reported that children from low SES groups showed a higher prevalence of excessive weight and obesity when compared to high SES groups [12,14,15]. Excessive weight and obesity is a worldwide epidemic, affecting developed and developing countries such as South Africa.

The prevalence of excessive weight gain and obesity in South Africa is currently following the same trend as the rest of the world, although it remains slightly lower. Various researchers have reported the prevalence of excessive weight in boys ranging from 6.4%–15.6%, while the prevalence of obesity has ranged between 3.2% and 10.9% [16,17,18,19,20,21]. This is in agreement with worldwide studies. Only a few longitudinal studies which explored the prevalence of excessive weight and obesity in South Africa could be found. One such study was conducted by Monyeki and colleagues [22] in the Ellisras District, Limpopo Province, on children aged between 3 and 10 years old. These researchers found that the risk of being overweight among primary school boys was 2.2%. Furthermore, they indicated that 14.5% of pre-school boys (between four and six years old) were overweight, though none were obese; however, the results changed dramatically for primary school boys (from 9 to 14 years old), with 24.6% being overweight and 1.2% obese [22]. Pienaar [23] also indicated that boys aged from six to nine years showed a high prevalence of obesity (3.2%). The Physical Activity and Health Longitudinal Study (PAHLS) on adolescents reported that an overall overweight/obesity prevalence of 15.4% was found and boys specifically reported an overweight/obesity prevalence of 10.3% [24].

In terms of race, culture and SES, South Africa is a diverse country. In the North West Province, the prevalence of overweight and obese boys ranged from 4.1% to 6.4% (overweight) and 1.5% to 4.0% (obese) [17,18,20,25]. Furthermore, these researchers reported that excessive weight and obesity were more prevalent among Caucasian boys (ranging between 11.6% and 13.3% vs. 2.6% and 6.4%) in comparison to African boys (ranging between 5.7% and 6.0% vs. 1.4% and 2.8%), all ranging between the ages of 6 and 13 years. Other researchers also reported the same tendency in Caucasian boys (ranging between 0.4% and 20.2% vs. 3.0% and 9.8%), representing a higher incidence of excessive weight gain and obesity when compared to African boys (ranging between 5.3% and 11.4% vs. 1.9% and 4.3%) in other provinces in South Africa [16,26,27,28].

SES plays an important role in South Africa. Several studies in South Africa found that the occurrence of excessive weight and obesity among boys between the ages of 1 and 10 years was lower (15.3% vs. 3.7%) in rural areas or lower SES groups [23,29,30,31] in comparison with high SES groups or urban areas (18.6% vs. 6.1%). The same tendency was found where children from lower SES groups (7.7% to 10%) had a lower occurrence of excessive weight gain and obesity compared to high SES groups (25.9% to 31.7%) in a longitudinal study in the North West Province among Grade 1 boys and six- to nine-year-old children [23]. This researcher also reported that the boys had a lower prevalence of excessive weight gain and obesity in 2010 (6.7% vs. 3.9%) when compared with 2013 (7.8% vs. 7.1%). From the above-mentioned literature, it can be observed that race and SES groups have a major effect on excessive weight gain and obesity in South Africa. It is further reported that excessive weight gain and obesity have a negative effect on academic performance [32,33,34].

Various studies in the USA, Germany and Canada have found an association between childhood (children aged between 7 and 16 years) weight gain or obesity and poor overall academic results (specific subjects are Mathematics, reading and science) [32,33,34,35,36,37,38,39]. These studies also found an association between weight and obesity, school attendance and lower cognitive abilities (visual perception, abstraction, memory, and concentration). Wang [40] also reports that changes in metabolism, related to obesity, can lead to cognitive restrictions, executive functioning delays, maths and reading delays, as well as attention deficits. Furthermore, Du Toit [41] explains the negative impact of obesity on academic skills through the relationship between an increased percentage of body fat and a decline in aerobic fitness. This relationship can further be explained by the process of brain activation; this process occurs as a result of a good level of physical fitness, which leads to an increase in blood circulation and a better delivery of oxygen to the brain [40,41,42]. In contrast with the above-mentioned studies, Falkner and colleagues [43] found results which indicated no observable pattern between poor academic performance and overweight or obese boys in Grade 7, 9 and 11 in Connecticut, USA. Longitudinal studies on pre-school (from four to six years old) and primary school (seven to thirteen years of age) children found a significant association between overweight children and academic test scores, especially mathematical test scores [44,45]. These researchers also found that overweight boys’ school life and attendance were negatively affected and that these children tended to achieve lower grades compared to boys of a normal weight. Furthermore, another longitudinal study (Avon Longitudinal Study of Parents and Children) reported that overweight and obese children (between 8 and 16 years old) attained a lower level of academic achievements compared to children of a normal weight [46]. Although Larsen’s [47] study focused on girls, it found that adolescent girls whose weight status changed over a three-year term from normal weight to overweight indicated a decrease in mathematical and reading ability. In comparison, Haywood and Pienaar [48] found no such difference in academic skills between girls of a normal weight and those experiencing obesity. Sirin [49] reported that SES has a positive and strong impact on a child’s academic performance. Various studies reported the same tendencies and further added that high SES groups achieved higher academic marks compared to middle and low SES groups [50,51]. Significant association between childhood overweight/obesity and academic performance was thus observed internationally (although gender differences were not the main focus), however, only a few of these studies [41] were conducted in South Africa and those that were made use of a cross-sectional design. However, it is important to study the long-term effects of excessive weight and obesity on academic performance to better understand their relationship. To the knowledge of the researchers, no published data regarding the effect of weight and obesity on academic performance specifically in primary school-age boys in South Africa are available.

It is clear that excessive weight gain and obesity are not just worldwide phenomena but also a localized problem that is experienced in South Africa. International statistics and South African statistics both indicate an increase in the prevalence of overweight and obese school children (especially boys). Excessive weight and obesity also have a severe impact on boys’ academic performance, however, to the researchers’ knowledge, there is no data to confirm or deny these findings among South African children. Only a few longitudinal studies have been conducted in this regard. Thus, the aim of this study was to determine the effect of excessive weight and obesity on academic performance on primary school boys in the North West Province of South Africa over a period of seven years, taking into account socio-economic status (SES).

## 2. Materials and Methods

### 2.1. Research Design

**Figure 1:**
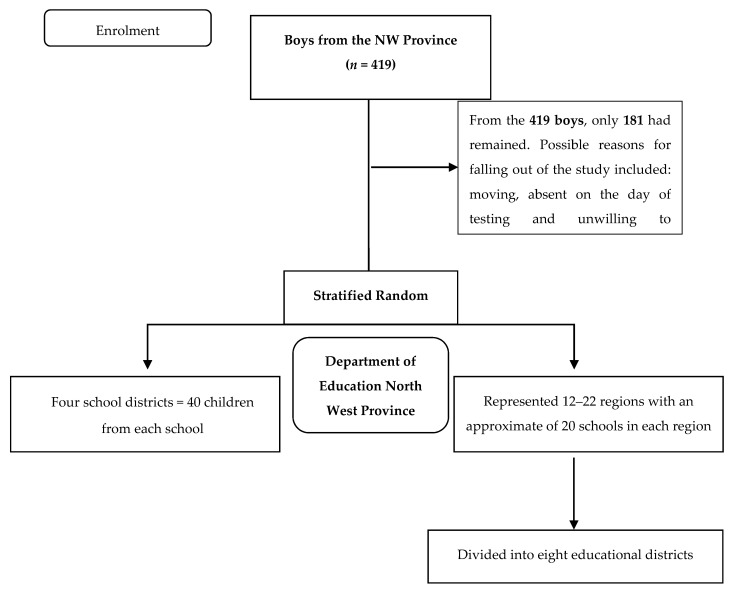
Flow diagram of the participants of this study.

The study formed part of a longitudinal research design, the North-West Child-Health-Integrated-Learning and Development (NW-CHILD) study and stretched over a period of seven years (2010–2016) which included a baseline measurement and two follow-up measurements. The baseline data was collected in 2010, the first follow-up measurement was conducted in 2013 and the second follow-up in 2016 across the different areas of the North West Province of South Africa. Only the data of the boys that participated in all three measurements was utilized for the purposes of this study (See Figure 1).

### 2.2. Research Group

**Figure 2:**
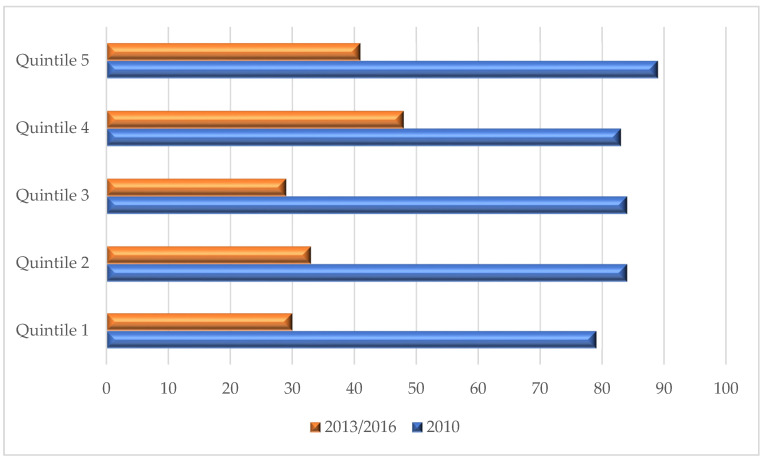
Participants according to the different Quintiles over the 7 years.

A total of 419 Grade 1 boys in the North West Province of South Africa initially participated in this study in 2010. This group consisted of Caucasian, African, Mixed race, and Indian boys. The boys were randomly selected from this list with regard to population density and school status, where Quintile 1 represented schools from poor SES areas and Quintile 5 represented schools from affluent SES areas. For the purpose of this study, Quintile 1 to 3 (*n* = 92) were grouped together as the low SES group and Quintile 4 to 5 (*n* = 89) as the high SES group. During the first follow-up measurements in 2013, only 282 of the participants were available. The last follow-up measurements were conducted in 2016, with the participants mostly comprised of learners in Grade 7, however, few of the participants were in Grade 6 because of retention. This group consisted of 181 (63 Caucasian and 118 African and Mixed race) boys. Furthermore, these boys were divided into Quintiles, with 30 of the boys in Quintile 1, 33 in Quintile 2, 29 in Quintile 3, 48 in Quintile 4 and 41 in Quintile 5 (please see Figure 2 for the distribution of the participants). Despite a large fall-out number from 2010 to 2016, statistical results still proved suitable for further analysis. For a complete discussion on methods for the NW-CHILD study, please refer to Kemp and colleagues [17].

### 2.3. Ethical Approval

Ethical approval for the execution of the project was obtained from the Ethics Committee of the North-West University, Potchefstroom Campus (No. NW-00070-09-A1, 2015/10/12). Permission to gather data during school hours was also obtained from the Department of Basic Education. Permission was also obtained from the principals of the schools which participated in the study. The parents or legal guardians of learners who participated in the study were asked to complete the informed consent forms whilst learners gave assent on the test-day. An effort was made to re-evaluate the same learners who had been evaluated in Grade 1 again during the two follow-up measurements. The data were collected by senior researchers and postgraduate students with a qualification in Human Movement Science, specializing in Kinderkinetics.

### 2.4. Measuring Instruments

Before any of the measurements were taken, the postgraduate students in Human Movement Sciences, specializing in Kinderkinetics, were trained to part take in the data collection. The researchers from the North-West University conducted the research. All of the researchers had an Kinanthropometry certification of Level 2.

### 2.5. Body Composition

The anthropometric measurements included body mass (kg), height (cm), waist circumference (cm) and three skinfolds (sub-scapular, triceps, and medial calf) (mm). The anthropometrist took anthropometric measurements in each year throughout the study to ensure quality and confirm inter-tester reliability, in accordance with the protocol of the International Society for the Advancement of Kinanthropometry [52]. Height was measured with a portable stadiometer to the nearest 0.1 cm and body mass was measured to the nearest 0.1 kg with an electronic scale (BF 511, Omron, Kyoto, Japan). Three skinfolds were taken, namely the sub-scapular (two centimeters from the sub-scapular landmark, running obliquely and laterally downwards), the triceps (is a parallel skinfold, taken along the long axis of the upper arm at the triceps skinfold site and the medial calf (a vertical measurement on the medial calf skinfold site) [52]. These skinfolds were measured with a pair of Harpenden skinfold calipers and each was measured twice to calculate an average measurement that was used for analysis. Each participant’s body mass index (BMI) [(kg/height (m)^2^] was calculated from the individual height and body mass measurements. The cut-off for indicating overweight was 21.9 kg/m^2^, whilst for obesity the cut-off was 27.76 kg/m^2^ [53]. The prevalence of excessive weight and obesity was determined by using the International age- and gender-specific cut-off points provided by Cole and colleagues [53] (see Table 1). Boys have a risk of being overweight (85th), obese (95th) and severely obese (98th) when the BMI is in the mentioned percentile for age and gender. A BMI of 35 kg/m^2^ was used in this study to determine severe obesity.

### 2.6. Scholastic Achievement

In Grade 1 (2010), during the June examinations, “The Mastery of Basic Learning Areas Questionnaire” was used to determine the academic performance of the different learning areas. A four-point scale was used: (1) indicating not achieved, (2) partially achieved, (3) achieved and (4) outstanding achievement. Lastly, a cluster point was calculated from three variables: reading, writing and mathematics. In Grade 4 (2013), the June examination results were collected from all participating schools and the same procedure was followed in 2016, when most participants had reached Grade 7. According to the Department of Basic Education’s Curriculum and Assessment Policy Statements (CAPS), the learning areas are: Mathematics, Home Language, Second Additional Language, Life Orientation (LO), Natural Science (NS), Social Science Technology, Creative Arts and Economic Management Science [54]. Academic performances regarding Annual National Assessments (ANA) of September 2010 and 2013 were collected from all the learners who participated in this study. The ANA results of 2016 were also collected after the learners had completed the ANA examination in September 2016. The Language and Mathematics results of the learners were made available by the Department of Basic Education [54]. All learning areas mentioned were assessed with the ANA grading scales (See Table 2). The rating code and percentages were used according to the ANA assessments.

### 2.7. Statistical Analysis

During this study, STATISTICA StatSoft [55] was used to analyze the data. Firstly, descriptive data was analyzed, and means and standard deviations were calculated. Repeated Measures ANOVA were used for data over time to determine the difference between the different socio-economic status (SES) and the boys’ body composition (BMI), as well as their academic performance (2010–2016). Two-way tables were used to determine any relationships and changes that may have occurred over time regarding overweight, obesity, SES and to compare the classifications of the different quintiles. Pearson Chi-square was used to indicate the significance of these associations (BMI and academic performance) and the level of statistical significance was set at *p* ≤ 0.05. The strength of the relationship was indicated by phi-coefficient, with *w* ≈ 0.1 indicating a small effect, *w* ≈ 0.3 a medium effect and *w* ≥ 0.5 a large effect. Lastly, Spearman rank order correlations between BMI and academic performance were determined. The strength of the correlation was set at *r* ≈ 0.1, indicating a small effect, *r* ≈ 0.3, indicating a medium effect and *r* ≈ 0.5, indicating a large effect [56].

## 3. Results

The group of boys (*n* = 181) consisted of 63 Caucasian and 118 African and Mixed-race boys (the last two groups were combined to achieve a smaller sample size). Table 3 reports descriptive statistics for 2010, 2013 and 2016, with regard to age, stature, weight and BMI. Table 3 reports that boys’ stature and BMI gradually increased with every measurement (from 2010 to 2016). This table also reports that the weight had a greater increase between 2013 (M = 32.6) and 2016 (M = 45.3), compared to 2010 (M = 23.1).

Two-way tables were used to determine the changes that might have occurred over time between SES and BMI. Table 4 reports that, in 2010, the high SES group had higher levels of excessive weight (termed overweight) (17.98%) and obesity (4.49%) when compared to the low SES group. The same tendency was found in 2013 and 2016, however, more boys were obese in 2013 (12.36%) among the high SES group when compared to 2010 (4.49%). Furthermore, in 2016, the high SES group contained more boys who were overweight (23.06%) compared to 2010 and 2013. Table 4 displays that, in 2013, one less overweight boy was reported when compared to 2010; however, the overweight percentage was higher in 2016 when compared to 2013. Furthermore, the percentage of boys of a normal weight decreased between 2010 and 2013. The same tendency was reported from 2013 to 2016. Lastly, obesity showed an increase between 2010 and 2013; however, a decrease was observed from 2013–2016. Statistical and practical significance was found among the high SES group (*p* = ≤0.001 and *w* = 0.28) and obese boys in 2010, in 2013 (*p* = ≤0.001 and *w* = 0.34) and 2016 (*p* = ≤0.001 and *w* = 0.35).

Repeated measure ANOVA’s were used to determine the longitudinal effect of BMI and academic performance, taking into consideration SES. Table 5 shows a statistical significance in 2010 between numeracy (*p* = 0.03), reading (*p* = 0.03), writing (*p* = 0.05) and the average academic mark (*p* = 0.02) with SES. In 2013, only one subject reported a statistical significance with SES, namely English (*p* = 0.02). Various subjects in 2016 reported a statistical significance with SES: Mathematics (*p* = 0.02), average academic mark (*p* = 0.001), First Additional Language (department) (*p* = 0.002), Mathematics (department) (*p* = 0.02) and average academic mark (department) (*p* = 0.001). Furthermore, only one subject reported a statistical significance with SES and BMI: First Additional Language (department) (*p* = 0.04). Effect sizes were further determined to establish the effect among normal weight, overweight, obesity and SES. Reading was the only subject which reported a medium effect (*d* = 0.52) among normal weight boys and SES. The same was reported in 2013, where only one subject reported a medium effect (*d* = 0.57), however, this time between Mathematics and SES. In 2016, Mathematics and Mathematics (department) reported a large effect among normal weight (*d* = 0.84 and *d* = 0.87 respectively). Furthermore, a medium effect (*d* = 0.55) was reported between Home Language (department) and normal weight. The same tendency was reported among the average academic mark and obesity (*d* = 0.57). Lastly, a small effect size was reported between Home Language and overweight boys (*d* = 0.26).

Lastly, Spearman Correlations were used to determine the relationship between academic performance and BMI. Table 6 indicates a positive correlation between BMI and academic subjects. Statistical (*p* < 0.05) and small (*r* ≥ 0.1) to medium (*r* ≥ 0.3) practical significant correlations were found between most of the subjects and BMI of the boys. Only Afrikaans (2013) showed no positive correlation (*r* = −0.06) with BMI. Table 6 also indicates that boys with a higher BMI value performed better in their academic subjects when compared to their other peers. In 2013, the Language (ANA), English and BMI association were much higher, with a medium (*r* ≥ 0.3) practical effect, when compared to 2010 and 2016. All the other subjects indicated a small (*r* ≥ 0.1) practical significance.

## 4. Discussion

The aim of this study was to determine the effect of excessive weight and obesity on the academic performance of primary school boys in the North West Province of South Africa over a period of seven years (2010–2016), taking SES into account.

In this study, it has become clear that the BMI values of the boys increased over a period of seven years (2010–2016) from 15.7 in 2010 to 17.5 in 2013 and 19.3 in 2016. These findings are consistent with the results of 25 other countries (school-aged children) such as North America (USA and Canada), South America (Brazil and Chile), Western Pacific region (Japan and Australia) and Europe (Finland, Germany, Spain and Greece) [57]. The same tendency was also reported by Zhang and Wang [57] in Shandong, China, and similarly in Thailand [58]. A supporting study, the IDEFICS (Identification and prevention of Dietary- and lifestyle-induced health Effects in Children and infants study), conducted in Spain, Cyprus, Sweden, Belgium, Germany, Hungary, Italy and Estonia found that the overall prevalence of excessive weight and obesity was higher among the following countries: Italy (42.4%), Cyprus (23.4%) and Spain (21.2%), whereas Belgium (9.4%) and Sweden (11.0%) had the lowest prevalence of an overweight and obese population [4]. These results support the current findings as most of these countries experience different socio-economic challenges, similarly to South Africa [4]; however, none of these studies were conducted in African countries. These findings strengthen this study as none of them are longitudinal and this study specifically focused on the changes that occurred over time (seven years).

The incidence of excessive weight and obesity in this study between the different SES groups indicate that boys from higher SES groups were more likely to be overweight (ranging from 17.98% to 23.60%) and obese (ranging from 4.49% to 8.99%) when compared to low SES groups (overweight: 1.09%–2.17% and obesity: 2.17%–4.35%). This study showed that the percentage of overweight boys increased with 5.62% from 2010 (17.98%) to 2016 (23.60%) among the high SES group while the boys from the low SES groups showed a consistent percentage (2.17%). Furthermore, this study reveals that the obesity percentages have increased nearly 8%, from 2010 (4.49%) to 2013 (12.36%). However, from 2013 (12.36%) to 2016 (8.99%), a 3.35% decrease in obesity among boys from the high SES groups was observed, whereas the boys from the low SES groups reported an increase in obesity from 2010 (2.17%) to 2013 (4.35%). The results further indicate that, even though there was a decrease in the obesity in the high SES boys, their prevalence was still higher than boys from low SES. The findings in this study may be due to the high SES groups having more access to food and/or having the financial support to purchase food, compared to the low SES groups. Furthermore, the slight decrease in overweight participants among the high SES groups might have been due to more physical activity or knowledge regarding healthy living. These findings correlate with other South African studies regarding boys from high SES groups who tend to report higher weight and obesity levels when compared to low SES groups [23,31]. Steyn et al. [31] and Pienaar [23] found similar results, also reporting that lower SES groups had lower BMI values when compared to high SES groups. This trend is in line with developing countries, where higher SES are usually associated with higher BMI levels.

The repeated measure ANOVA reported that SES had a greater significance (r = 0.74) when associated with academic subjects. During the first measurement in 2010, all subjects (numeracy, reading and writing) reported a significance regarding SES. In 2013, only English showed any significance, while in 2016, Mathematics, average academic score, First Additional Language (department), Mathematics (department) and average academic marks reported significance with regard to low SES. To confirm the findings of a significant association between academic performance and SES, Caro et al. [59] reported that low SES has a major effect on academic performance, especially Mathematics. The same tendency was reported among primary school boys in the United States of America [49,51] and the Ganderbal District in India [50]. Lastly, concerning the academic performance and the effect that BMI might have on it, it was clear that the boys with higher BMI values performed better in Language and Mathematic subjects. In 2010, a positive correlation with a small effect (*r* = 0.1) was observed in all three major assessment areas (literacy, numeracy and reading) and BMI. The same tendency was observed in 2013, where nearly all the subjects reported a small effect with a positive correlation (*r* = 0.1). Language (as tested with the ANA test) and English were the only two subjects that had a positive correlation with a medium effect (*r* = 0.3). However, Afrikaans is the only subject that reported no correlation (*r* = −0.06) with regard to BMI values. Furthermore, in 2016, all the subjects reported a small effect (*r* = 0.1). The results of this study are in contradiction with other studies [32,33,35,36,37,38,39,44,45,46] with regard to overweight and obese children (in this study, boys) where the results reported that the higher the BMI value the better academic the performance at school. Supporting our findings, a study in Connecticut, USA [43] and Kuwait [60,61], found that overweight or obese boys had no significant association with academic test scores. Baxter and colleagues [62] also reported in their study in Columbia, South Carolina, that no significant association was found between BMI and poor academic performance; however, an association between academic performance and SES were reported. Furthermore, a study by Shah and Maiya [63] in the Anand District reported that boys with high BMI values usually exhibited a lower academic performance. Confirming the last-mentioned study in Saudi Arabia, on the same age group, no correlation between BMI and school performance was found [64].

## 5. Strengths and Limitations

The strength of this study is the longitudinal design and nature of the analyses that were used. This study included 181 primary school boys that were randomly selected and that were representative of the South African population. The study was conducted throughout the primary school years of the participants, stretching over 7 years. According to the researchers, no studies have previously been conducted that focused on the individual school subjects or the academic achievements over a long-term period. However, like all studies, this study had certain limitations that could have affected the results and the authors would like to highlight these. A significant dropout rate over the two follow-up periods of the study may have affected the generalization of the findings. Furthermore, the tool that was used for measuring SES was the quintile schools and as children commute to and from schools this may not be the best representation of SES status.

## 6. Conclusions

This study was conducted over the course of seven years (2010–2016), thus being one of the few longitudinal studies in South Africa, regarding weight, obesity and academic performance, whilst also taking SES into consideration. The study reported that primary school boys with high BMI values (thus being overweight or obese) achieved better academically compared to boys of a normal weight; however, BMI is not the only factor contributing to academic performance, as SES also had an influence. In this study, it is found that SES had a greater influence on academic performance compared to BMI. Boys from low SES groups exhibited a lower academic performance compared to boys from high SES groups. The study also found only one subject that reported to have significance in relation to BMI and SES. This study reported valuable findings. Due to the longitudinal effect of the study, which followed the boys throughout their primary school career, valuable data was obtained with regard to weight and obesity and academic performance. However, more studies are recommended in all nine provinces across South Africa as the current study only focused on the North West Province. As observed in the literature, excessive weight and obesity affect academic performance; however, the findings reported the contrary. It is thus recommended that further studies be conducted, especially longitudinal studies, in the South African setting regarding weight, obesity and academic performance, as well as taking the parents financial income and education into account, to support the findings.

## Figures and Tables

**Table 1:** Age specific BMI cut-off points for overweight and obesity [53].

	Body Mass Index: Overweight	Body Mass Index: Obesity
Age (yrs.)	Boys	Boys
6	17.55	19.78
6.5	17.71	20.23
7	17.92	20.63
7.5	18.16	21.09
8	18.44	21.60
8.5	18.76	22.17
9	19.10	22.77
9.5	19.46	23.39
10	19.84	24.00
10.5	20.20	24.57
11	20.55	25.10
11.5	20.89	25.58
12	21.22	26.02
12.5	21.56	26.43
13	21.91	26.84
13.5	22.27	27.25

**Table 2:** Grading scales for ANA assessments.

Rating Code	Description of Competence	Percentage
7	Outstanding achievement	80–100
6	Meritorious achievement	70–79
5	Substantial achievement	60–69
4	Adequate achievement	50–59
3	Moderate Achievement	40–49
2	Elementary achievement	30–39
1	Not achieved	0–29

**Table 3:** Descriptive statistics from 2010 to 2016.

Variable	N	Mean	Minimum	Maximum	SD
2010					
Age	181	6.9	6.0	7.8	0.4
Stature	181	120.8	102.1	141.7	6.5
Weight	181	23.1	16.4	45.9	5.0
BMI	181	15.7	12.6	26.8	2.2
2013					
Age	181	9.9	9.0	10.7	0.4
Stature	181	136.1	117.0	161.0	6.9
Weight	181	32.6	19.6	65.6	8.8
BMI	181	17.5	12.9	30.8	3.4
2016					
Age	181	12.9	11.9	13.7	0.4
Stature	181	152.6	131.9	178.8	9.2
Weight	181	45.3	23.1	100.8	13.5
BMI	181	19.3	14.0	36.5	4.2

N = number of participants; SD standard deviation, BMI = Body Mass Index.

**Table 4:** Two-way frequency table for socio-economic status (SES) and BMI categories over time (2010–2016).

	Normal	Overweight	Obese	Overall
2010				
Low SES				
N	88	2	2	92
%	95.65	2.17	2.17	50.83
High SES				
N	69	16	4	89
%	77.53	17.98	4.49 ^▲^^#^	49.17
Overall				
N	157	18	6	181
%	86.74	9.94	3.32	100
2013				
Low SES				
N	87	1	4	92
%	94.57	1.09	4.35	50.83
High SES				
N	62	16	11	89
%	69.66	17.98	12.36 ^▲^^#^	49.17
Overall				
N	149	17	15	181
%	82.30	9.40	8.30	100
2016				
Low SES				
N	86	2	4	92
%	93.48	2.17	4.35	50.83
High SES				
N	60	21	8	89
%	67.42	23.60%	8.99 ^▲^^#^	49.17
Overall				
N	146	23	12	181
%	80.66	12.71	6.63	100

^▲^ *p* ≤ 0.01; ^#^ w ≥ 0.3; SES = socio-economic status, N = Number of participants, % = percentage.

**Table 5:** Repeated measure ANOVA per subject, per year from 2010–2016.

2010	**Subjects**	**Normal**		**Overweight**		**Obesity**		**MSE**	***p*-Values**			**Effect Size of SES**		
		**Low SES**	**High SES**	**Low SES**	**High SES**	**Low SES**	**High SES**		**BMI**	**SES**	**BMI & SES**	**Normal**	**Over-weight**	**Obesity**
	Numeracy	69.32	83.33	62.50	89.06	87.50	93.75	337.4	0.21	0.03 *	0.59	0.76	1.45	0.34
	Reading	62.22	73.19	50.00	85.94	75.00	81.25	447.5	0.54	0.03 *	0.29	0.52▲	1.70	0.30
	Writing	67.61	74.28	62.50	89.06	75.00	87.50	398.9	0.43	0.05 *	0.42	0.33	1.33	0.63
	Average	66.38	76.93	58.33	88.02	79.17	87.50	324.1	0.34	0.02 *	0.38	0.59	1.65	0.46
2013	English	42.48	67.45	58.00	65.50	47.50	58.18	247.8	0.63	0.02 *	0.20	1.59	0.48	0.68
	Mathematics	56.52	65.57	57.00	69.19	48.25	60.82	256.1	0.39	0.08	0.92	0.57▲	0.76	0.79
	Language (ANA)	41.68	69.50	-	67.75	40.00	64.91	271.5	0.58	-	0.79	1.69	-	1.51
	Mathematics (dep.)	39.36	59.70	80.00	65.25	47.33	53.82	308.8	0.05	0.58	0.09	1.16	0.84	0.37
2016	Home Language	56.99	63.34	56.50	59.95	54.25	54.43	178.4	0.39	0.45	0.75	0.48	0.26•	0.01
	1st Additional Language	49.02	63.44	47.00	64.40	60.25	52.86	222.5	0.99	0.10	0.08	0.97	1.17	0.50
	Mathematics	44.20	58.00	48.00	59.70	36.25	47.71	269.1	0.20	0.02 *	0.96	0.84■	0.71	0.70
	Average	49.29	62.83	47.22	63.45	48.10	55.21	126.6	0.48	0.001 *	0.64	1.20	1.44	0.63
	Home Language (dep.)	51.36	59.42	47.99	61.80	52.00	49.97	217.3	0.66	0.17	0.49	0.55▲	0.94	0.14
	1st Add. Language (dep.)	44.52	56.77	24.00	62.26	51.50	50.54	250.2	0.46	0.002 *	0.04	0.77	2.42	0.06
	Mathematics (dep.)	41.03	57.71	38.50	58.73	36.75	43.19	364.0	0.32	0.02 *	0.68	0.87■	1.06	0.34
	Average	41.80	58.00	33.33	60.85	41.54	47.72	117.9	0.28	0.001 *	0.13	1.49	2.53	0.57▲

* *p* ≤ 0.05; SES- socio-economic status; MSE = Mean Square Error, BMI = Body Mass Index, • small effect size; ▲ medium effect size; ■ large effect size; 1st Add. Language (dep) = First Additional Language (department); dep.—department, ANA = Annual National Assessment.

**Table 6:** Spearman rank order correlations on academic performance (per subject) and BMI.

Year	Academic Subjects	High BMI
1 (2010)	ANA Numeracy (%)	0.19 *^,#^
	ANA Reading (%)	0.17 *^,#^
	ANA Writing (%)	0.19 *^,#^
	Average	0.21 *^,#^
2 (2013)	Language (ANA)	0.30 *^,##^
	Mathematics (%)	0.28 *^,#^
	Afrikaans	−0.06
	English	0.31 *^,##^
	Mathematics	0.16 *^,#^
	Average	0.28 *^,#^
3 (2016)	ANA Home Language	0.13 ^#^
	ANA First Additional Language	0.28 *^,#^
	ANA Mathematics	0.20 *^,#^
	Average	0.31 *^,##^
	Home Language (department)	0.16 *^,#^
	First Additional Language (department)	0.29 *^,#^
	Mathematics (department)	0.27 *^,#^
	Average	0.37 *^,##^

* *p* < 0.05, BMI = Body Mass Index, ANA = Annual National Assessment, % = Percentage, Practical significance: *r* = 0.1 ^#^ small effect, *r* = 0.3 ^##^ medium effect; BMI—Body Mass Index.

## Data Availability

The dataset is the property of the North-West University under supervision of Anita E Pienaar. In this regard, A.E. Pienaar should be contacted if, for any reason, the data included in this paper needs to be shared. A.E. Pienaar is the Principal investigator of this study and gave permission that we can use the data.
